# Association between neurodevelopmental impairments and motor function in Duchenne muscular dystrophy

**DOI:** 10.1002/acn3.51914

**Published:** 2023-10-07

**Authors:** Mathula Thangarajh, Michael P. McDermott, Michela Guglieri, Robert C. Griggs

**Affiliations:** ^1^ Department of Neurology Virginia Commonwealth University Richmond Virginia USA; ^2^ University of Rochester Medical Center School of Medicine and Dentistry Rochester New York USA; ^3^ John Walton Muscular Dystrophy Research Centre Newcastle University and Newcastle Hospitals National Health Service Foundation Trust Newcastle UK

## Abstract

**Objective:**

We explored various prognostic factors of motor outcomes in corticosteroid‐naive boys with Duchenne Muscular Dystrophy (DMD).

**Methods:**

The associations between parent‐reported neurodevelopmental concerns (speech delay, speech and language difficulties (SLD), and learning difficulties), *DMD* mutation location, and motor outcomes (6‐minute walk distance (6MWD), North Star Ambulatory Assessment (NSAA) total score, 10‐meter walk/run velocity, and rise from floor velocity) were studied in 196 corticosteroid‐naive boys from ages 4 to less than 8 years.

**Results:**

Participants with SLD walked 25.8 fewer meters in 6 minutes than those without SLD (*p* = 0.005) but did not demonstrate statistical differences in NSAA total score, 10‐meter walk/run velocity, and rise from floor velocity. Participants with distal *DMD* mutations with learning difficulties walked 51.8 fewer meters in 6 minutes than those without learning difficulties (*p* = 0.0007). Participants with distal *DMD* mutations were slower on 10‐meter walk/run velocity, and rise from floor velocity (*p* = 0.02) than those with proximal *DMD* mutations. Participants with distal *DMD* mutations, who reported speech delay or learning difficulties, were slower on rise from floor velocity (*p* = 0.04, *p* = 0.01) than those with proximal *DMD* mutations. The mean NSAA total score was lower in participants with learning difficulties than in those without (*p* = 0.004).

**Interpretation:**

Corticosteroid‐naive boys with DMD with distal *DMD* mutations may perform worse on some timed function tests, and that those with learning difficulties may perform worse on the NSAA. Pending confirmatory studies, our data underscore the importance of considering co‐existing neurodevelopmental symptoms on motor outcome measures.

## Introduction

Dystrophin—the protein product of the dystrophin gene (*DMD)*—plays vital roles as a membrane scaffold and stabilizer in skeletal and cardiac muscles, and in synaptogenesis and cell signaling pathways in the brain.^1^, ^2^, ^3^, ^4^, ^5^ Mutations in *DMD* cause Duchenne Muscular Dystrophy (DMD), an X‐linked multi‐system disease characterized by progressive skeletal muscle wasting and weakness, diaphragmatic weakness, heart failure, neurodevelopmental co‐morbidities, and cognitive impairments.^6^ In the brain, full‐length and shorter dystrophin isoforms are transcribed from unique exons. Distal *DMD* mutations including nucleotides 3’ *DMD* as well as *DMD* intron 44 have been shown to affect the expression of shorter dystrophin isoforms (Dp140, Dp116, and Dp71).^7^

In recent years, there has been greater appreciation of the neurodevelopmental symptoms in DMD. Rates of autism spectrum and attention‐deficit hyperactivity disorders are 3 to 4 times higher in DMD than in the general population^7^, ^8^, ^9^, ^10^, ^11^, ^12^, ^13^, ^14^, ^15^; these symptoms affect infant‐parent bonding, schooling, and social relationships.^16^ Nearly 100% of boys with DMD have impairment in cognitive flexibility, big‐picture thinking and planning, collectively called executive function,^7^ which contribute to nearly 50% of developmental gains in intellectual function during childhood.^17^ Not surprisingly, parents report that these cumulative neurodevelopmental symptoms interfere with schooling, peer relationships, and development of self‐efficacy skills.^16^ Further, we and others have shown that speech delay is more common in DMD than in the general population.^13^, ^14^, ^19^ Our prior work from Finding the Optimal Regimen for Duchenne Muscular Dystrophy (FOR‐DMD) study showed that 38% of the enrolled boys reported speech delay, and this symptom was more common in boys with distal *DMD* mutations compared to those with proximal *DMD* mutations.^19^

Co‐existing neurodevelopmental symptoms in DMD appear to be associated with worse long‐term motor, respiratory, and cardiac outcomes. A retrospective study analyzed data from 75 corticosteroid‐naive boys with DMD followed longitudinally over a 10‐year period from a single national neuromuscular referral hospital.^18^ A subgroup of boys (*n* = 15) whose initial presenting symptom of DMD was “psychomotor delay” walked independently later (mean 20 months, SD 7.9 months) and lost ambulation earlier (mean 9 years, SD 1.6 years) than the subgroup of boys who presented with “pure motor delay” (*n* = 16); the latter group walked independently at a mean of 15 months (SD 3.8 months) and lost ambulation at a mean of 12.6 years (SD 2 years). Likewise, both cardiac and respiratory declines occurred earlier in the subgroup presenting with “psychomotor delay.” Five of the 15 boys in this subgroup had a left ventricular ejection fraction of less than 55% *prior* to 10 years of age, compared to none of the boys in the “pure motor delay” subgroup. In addition, mean forced vital capacity was 65% at 10 years of age in the subgroup presenting with “psychomotor delay,” compared to 80% at 10 years of age in the subgroup presenting with “pure motor delay.”

In this study, we explored whether neurodevelopmental concerns—namely speech and language delay (SLD), speech delay, and learning difficulties—are prognostic of *pretreatment* motor function in DMD. We postulated that young corticosteroid‐naive boys with neurodevelopmental concerns *or* with distal *DMD* mutations would demonstrate worse performance on motor function tests than those without neurodevelopmental concerns or with proximal *DMD* mutations.

## Methods

### Study design and participants

The FOR‐DMD trial enrolled 196 corticosteroid‐naive boys between the ages of 4 and <8 years in five countries with the aim of comparing three different corticosteroid regimens with respect to efficacy and safety. Detailed information on the rationale and study design has been previously published.^20^ Briefly, the inclusion criteria for the FOR‐DMD trial were as follows: corticosteroid‐naive boys with genetically‐confirmed *DMD* mutation, ages 4 years to less than 8 years, able to arise independently from floor and able to provide reproducible forced vital capacity (FVC) measurements, as well as parent or guardian able to give written consent and comply with study visits and drug administration plan. The study was conducted in accordance with the Declaration of Helsinki (2000) and the Principles of Good Clinical Practice. Written informed consent was obtained from all parents/legal guardians of the study participants prior to commencement of study procedures. This clinical trial is registered under ClinicalTrials.Gov (NCT01603407).

### Study measures

In this study, neurodevelopmental concerns were defined broadly. We extracted parent‐reported concerns of SLD and the age at which the child first spoke in full sentences (language acquisition) from the medical history form completed at the screening visit. SLD was queried as present, absent, or unknown. Speech delay was defined to be present if the parent had reported that the age of first speaking in full sentences was later than 42 months.^21^ Learning difficulties were reported as present, absent, or unknown by parent. Motor function outcomes obtained at the baseline visit (or screening visit if the value at the baseline visit was missing) included six‐minute walk distance (6MWD), North Star Ambulatory Assessment (NSAA) total score, 10‐meter walk/run velocity, and rise from floor velocity. All of these tests were administered and recorded by a trained study‐team physical therapist.

### *DMD* mutation data

*DMD* mutation data were available for 193 of 196 participants who were categorized as having proximal *DMD* (proximal to 5’ *DMD* intron 44) or distal *DMD* (mutations in 3’ *DMD* including intron 44) mutations.

### Statistical analysis

For each of the motor function outcomes, three analyses of covariance models were fit, one that included SLD, a second that included speech delay, and a third that included learning difficulties; all models included *DMD* mutation and age. These models were used to estimate differences in adjusted mean outcome between boys with distal versus proximal *DMD* mutations, between boys with and without SLD, between boys with and without speech delay, and between boys with and without learning difficulties. The interactions between *DMD* mutation type and either SLD, speech delay, or learning difficulties were examined by adding the respective interaction term to the appropriate model. Because the models with SLD included larger sample sizes than the models with speech delay and learning difficulties, the results concerning differences between those with distal versus proximal *DMD* mutations are interpreted using the former model. Due to the exploratory nature of the analyses, no corrections were performed for multiple comparisons unless an interaction was identified, in which case subgroup comparisons incorporated a Tukey–Kramer adjustment.

## Results

### Demographic and clinical characteristics

A total of 196 participants were enrolled in the FOR‐DMD trial. The mean age at time of baseline motor function assessment was 5.8 years (SD 1.0) were enrolled in the FOR‐DMD study. Parent‐reported SLD was reported in 75 of 195 participants (38%; data missing in 1 participant), and speech delay was reported in 18 of 167 participants (11%; data missing in 29 participants). In the 167 participants with available data on speech and language acquisition, speech delay was reported in 16 of the 67 participants with SLD, and SLD was reported in 16 of the 18 with speech delay. Learning difficulties were reported in 50 of 181 boys (28%). Among the 48 participants for whom the severity of learning difficulties was reported, the severity was mild in 69% (33/48), moderate in 29% (14/48), and severe in 2% (1/48). The mean age of participants with and without SLD, and with and without speech delay was the same (5.8 years). The mean ages of participants with and without learning difficulties were 6.2 and 5.8 years, respectively. The numbers of participants with proximal *DMD* versus distal *DMD* mutations were 88 and 105, respectively. The mean ages of participants with proximal versus distal *DMD* mutations were 5.9 and 5.8 years, respectively.

### 6MWD

**Figure 1:**
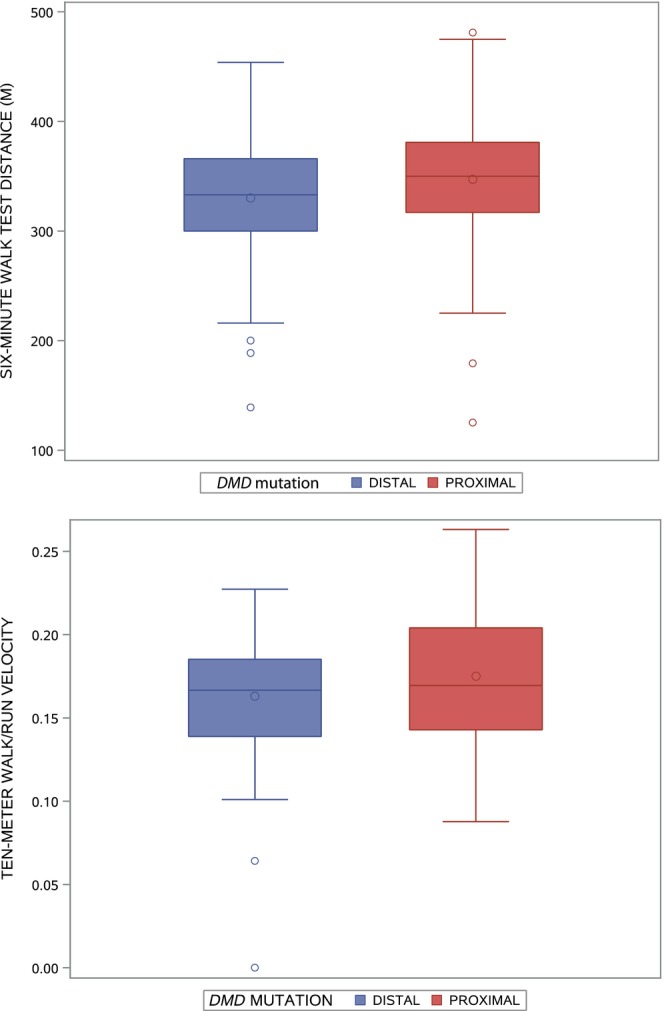
Boxplots of 6‐minute walk distance in meters by *DMD* mutation (proximal, distal) (left), and interaction between *DMD* mutation and learning difficulties (right). The line inside the box represents the median, and the circle inside the box represents the mean. The ends of the boxes represent the 25th and 75th percentiles of the distribution; the lines extending from the boxes indicate the range of the data, with the exception of outlier values (indicated by circles) that are more than (1.5 × interquartile range) from the nearest quartile.

Results of the analysis of covariance models for 6MWD are presented in Table 1. Those with SLD had an adjusted mean 6MWD of 319.4 m compared to 345.2 m in those without SLD (group difference = −25.8 m, 95% confidence interval [CI] ‐43.7 to −7.8, *p* = 0.005). This difference was consistent between those with distal (−25.1 m) and proximal (−26.8 m) *DMD* mutations (*p* = 0.93 for the interaction between SLD and *DMD* mutation type). The difference in adjusted mean 6MWD between those with distal versus proximal *DMD* mutations was −13.4 m (95% CI −30.9 to 4.2, *p* = 0.13; Fig. 1, left). The difference in adjusted mean 6MWD between those with and without speech delay was −16.6 m (95% CI −47.6 to 14.5, *p* = 0.29). In the model with learning difficulties, there was an interaction between learning difficulties and *DMD* mutation type (*p* = 0.03), with the adjusted mean 6MWD being lower in those with distal *DMD* mutations and learning difficulties (291 m) than in those in the other three groups (adjusted mean 6MWD ranging from 49.3 to 56.1; Table 1, Fig. 1, right).

**Table 1:** Associations between 6‐minute walk distance and *DMD* mutation, speech and language difficulties, speech delay, and learning difficulties.

(a) Model with *DMD* mutation, speech and language difficulties, and age							
Mean 6MWD (m)				Mean 6MWD (m)			
*DMD* Mutation		Group difference (95% CI)	*p*‐value	Speech and language difficulties		Group difference (95% CI)	*p*‐value
Distal	Proximal			Yes	No		
325.6	339.0	−13.4 (−30.9, 4.2)	0.13	319.4	345.2	−25.8 (−43.7, −7.8)	0.005

(b) Model with *DMD* mutation, speech delay, and age							
Mean 6MWD (m)				Mean 6MWD (m)			
*DMD* mutation		Group difference (95% CI)	*p*‐value	Speech delay^1^		Group difference (95% CI)	*p*‐value
Distal	Proximal			Yes	No		
317.7	336.4	−18.7 (−38.4, 1.0)	0.06	318.8	335.3	−16.6 (−47.6, 14.5)	0.29

(c) Model with *DMD* mutation, learning difficulties, interaction between *DMD* mutation and learning difficulties, and age				
	Group (*DMD* mutation/learning difficulties)			
	Distal/yes	Proximal/yes	Distal/no	Proximal/no
Mean 6MWD (m)	290.6	339.9	342.4	346.7
Difference vs. distal/yes group		49.3	51.8	56.1
95% CI for difference^2^		(1.6, 96.9)	(17.6, 86.0)	(22.8, 89.4)
*p*‐value^2^		0.04	0.0007	0.0001

6MWD, six‐minute walk distance; CI, confidence interval.

^1^ Speech delay was defined as age first speaking in full sentences >42 months.

^2^ Adjusted for all six possible pairwise group comparisons using the Tukey–Kramer method; differences among the proximal/yes, distal/no, and proximal/no groups were not statistically significant (*p* > 0.97).

### Ten‐meter walk/run velocity

**Figure 2:**
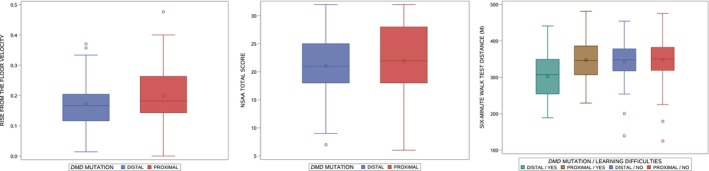
Boxplots of motor outcomes by *DMD* mutation. Ten‐meter walk/run velocity (left), rise from the floor time (middle), and NSAA (right). The line inside the box represents the median, and the circle inside the box represents the mean. The ends of the boxes represent the 25th and 75th percentiles of the distribution; the lines extending from the boxes indicate the range of the data, with the exception of outlier values (indicated by circles) that are more than (1.5 × interquartile range) from the nearest quartile.

Results of the analysis of covariance models for 10‐meter walk/run velocity are presented in Table 2. Differences in adjusted mean 10‐meter walk/run velocity between those with (1.72 m/sec) and without (1.67 m/sec) SLD (*p* = 0.34), and between those with (1.72 m/sec) and without (1.68 m/sec) speech delay (*p* = 0.68), were not significant. Those with a distal *DMD* mutation had an adjusted mean velocity of 1.63 m/sec compared to 1.76 m/sec in those with a proximal *DMD* mutation (group difference = −0.13 m/sec, 95% CI −0.24 to −0.02, *p* = 0.02; Fig. 2, left). This difference was slightly larger in those with SLD (−0.24 m/sec) than in those without SLD (−0.07 m/sec), but the interaction between the location of *DMD* mutation and SLD was not significant (*p* = 0.13). The difference in adjusted mean velocity between those with (1.60 m/sec) and without (1.72 m/sec) learning difficulties was −0.12 m/sec (95% CI −0.25 to 0.01, *p* = 0.07).

**Table 2:** Associations between 10‐meter walk/run velocity and *DMD* mutation, speech and language difficulties, speech delay, and learning difficulties.

(a) Model with *DMD* mutation, speech and language difficulties, and age							
Mean 10‐meter walk/run velocity (m/sec)				Mean 10‐meter walk/run velocity (m/sec)			
*DMD* mutation		Group difference (95% CI)	*p*‐value	Speech and language difficulties		Group difference (95% CI)	*p*‐value
Distal	Proximal			Yes	No		
1.63	1.76	−0.13 (−0.24, −0.02)	0.02	1.72	1.67	0.05 (−0.06, 0.17)	0.34

(b) Model with *DMD* mutation, speech delay, and age							
Mean 10‐meter walk/run velocity (m/sec)				Mean 10‐meter walk/run velocity (m/sec)			
*DMD* Mutation		Group difference (95% CI)	*p*‐value	Speech delay^1^		Group difference (95% CI)	*p*‐value
Distal	Proximal			Yes	No		
1.64	1.76	−0.12 (−0.24, 0.00)	0.05	1.72	1.68	0.04 (−0.15, 0.23)	0.68

(c) Model with *DMD* mutation, learning difficulties, and age							
Mean 10‐meter walk/run velocity (m/sec)				Mean 10‐meter walk/run velocity (m/sec)			
*DMD* mutation		Group difference (95% CI)	*p*‐value	Learning difficulties		Group difference (95% CI)	*p*‐value
Distal	Proximal			Yes	No		
1.61	1.71	−0.11 (−0.22, 0.01)	0.06	1.60	1.72	−0.12 (−0.25, 0.01)	0.07

CI, confidence interval.

^1^ Speech delay was defined as age first speaking in full sentences >42 months.

### Rise from the floor velocity

Results of the analysis of covariance models for rise from the floor velocity are presented in Table 3. Differences in adjusted mean rise from the floor velocity between those with (0.184 rise/sec) and without (0.187 rise/sec) SLD (*p* = 0.77), and between those with (0.161 rise/sec) and without (0.189 rise/sec) speech delay (*p* = 0.16), were not significant. Those with a distal *DMD* mutation had an adjusted mean velocity of 0.171 rise/sec compared to 0.199 rise/sec in those with a proximal *DMD* mutation (group difference = −0.028 rise/sec, 95% CI −0.052 to −0.005, *p* = 0.02; Fig. 2, center). This difference was slightly larger in those with SLD (−0.040 rise/sec) than in those without SLD (−0.022 rise/sec), but the interaction between *DMD* mutation type and SLD was not significant (*p* = 0.47). The difference in adjusted mean velocity between those with (0.169 m/sec) and without (0.189 m/sec) learning difficulties was −0.020 rise/sec (95% CI −0.047 to 0.006, *p* = 0.14).

**Table 3:** Associations between rise from the floor velocity and *DMD* mutation, speech and language difficulties, speech delay, and learning difficulties.

(a) Model with *DMD* mutation, speech and language difficulties, and age							
Mean rise from the floor velocity (rise/sec)				Mean rise from the floor velocity (rise/sec)			
*DMD* mutation		Group difference (95% CI)	*p*‐value	Speech and language difficulties		Group difference (95% CI)	*p*‐value
Distal	Proximal			Yes	No		
0.171	0.199	−0.028 (−0.052, −0.005)	0.02	0.184	0.187	−0.004 (−0.027, 0.020)	0.77

(b) Model with *DMD* mutation, speech delay, and age							
Mean rise from the floor velocity (rise/sec)				Mean rise from the floor velocity (rise/sec)			
*DMD* mutation		Group difference (95% CI)	*p*‐value	Speech delay^1^		Group difference (95% CI)	*p*‐value
Distal	Proximal			Yes	No		
0.162	0.188	−0.025 (−0.050, −0.001)	0.04	0.161	0.189	−0.028 (−0.067, 0.011)	0.16

(c) Model with *DMD* mutation, learning difficulties, and age							
Mean rise from the floor velocity (rise/sec)				Mean rise from the floor velocity (rise/sec)			
*DMD* Mutation		Group difference (95% CI)	*p*‐value	Learning difficulties		Group difference (95% CI)	*p*‐value
Distal	Proximal			Yes	No		
0.164	0.194	−0.030 (−0.054, −0.006)	0.01	0.169	0.189	−0.020 (−0.047, 0.006)	0.14

CI, confidence interval.

^1^ Speech delay was defined as age first speaking in full sentences >42 months.

### NSAA total score

Results of the analysis of covariance models for NSAA total score are presented in Table 4. No differences in adjusted mean scores were apparent between those with distal versus proximal *DMD* mutations (Fig. 2, right), between those with and without SLD, and between those with and without speech delay. The mean NSAA total score was lower in those with reported learning difficulties (19.5) than in those without learning difficulties (22.2) (group difference = −2.7, 95% CI −4.6 to −0.9, *p* = 0.004).

**Table 4:** Associations between North Star Ambulatory Assessment total score and *DMD* mutation, speech and language difficulties, speech delay, and learning difficulties.

(a) Model with *DMD* mutation, speech and language difficulties, and age							
Mean NSAA total score				Mean NSAA total score			
*DMD* mutation		Group difference (95% CI)	*p*‐value	Speech and language difficulties		Group difference (95% CI)	*p*‐value
Distal	Proximal			Yes	No		
21.0	21.7	−0.7 (−2.3, 0.9)	0.39	21.1	21.6	−0.5 (−2.2, 1.1)	0.51

(b) Model with *DMD* mutation, speech delay, and age							
Mean NSAA total score				Mean NSAA total score			
*DMD* mutation		Group difference (95% CI)	*p*‐value	Speech delay^1^		Group difference (95% CI)	*p*‐value
Distal	Proximal			Yes	No		
20.7	21.3	−0.6 (−2.4, 1.1)	0.48	20.6	21.3	−0.7 (−3.5, 2.1)	0.64

(c) Model with *DMD* mutation, learning difficulties, and age							
Mean NSAA total score				Mean NSAA total score			
*DMD* mutation		Group difference (95% CI)	*p*‐value	Learning difficulties		Group difference (95% CI)	*p*‐value
Distal	Proximal			Yes	No		
20.6	21.0	−0.4 (−2.0, 1.2)	0.61	19.5	22.2	−2.7 (−4.6, −0.9)	0.004

CI, confidence interval; NSAA, North Star Ambulatory Assessment.

^1^ Speech delay was defined as age first speaking in full sentences >42 months.

## Discussion

DMD is a disease caused by mutations in a single gene; yet, heterogeneity in clinical presentation, disease severity, and disease progression are well‐documented.^22^, ^23^, ^24^, ^25^, ^26^, ^27^, ^28^, ^29^, ^30^, ^31^ Further, genetic modifiers as well the beneficial effects of oral corticosteroids—the standard‐of‐care—in DMD^32^ significantly alter the trajectory of disease course. Given the resource‐intense nature of clinical trials in DMD, and several Phase 2/3 clinical trials failing to demonstrate a treatment effect on the primary outcome measure,^33^ better understanding of prognostic factors that contribute to disease heterogeneity can help not only clinical trial design but can inform patient care and management.

Our objective with this study was to explore prognostic factors of motor outcomes related to broadly‐defined neurodevelopmental concerns (SLD, speech delay, learning difficulties) as well as *DMD* genotype in corticosteroid‐naive boys with DMD. We found that those with SLD walked an average of 26 *fewer* meters in the 6MWD compared to those without SLD but there were no significant differences between those with and without SLD with respect to NSAA total score and timed motor function tests. With regard to *DMD* mutation location and motor outcomes, those with distal *DMD* mutations walked an average of 19 fewer meters in the 6MWD compared to those with proximal *DMD* mutations, and were slower in 10‐meter walk/run velocity and rise from the floor velocity, but did not demonstrate significant differences in NSAA total score compared to those with proximal *DMD* mutations. With respect to learning difficulties and functional outcomes, we observed that those with distal *DMD* mutations and learning difficulties walked an average of 49–56 *fewer* meters in the 6MWD than those with proximal *DMD* mutations or no learning difficulties. Also, those with learning difficulties had a lower mean NSAA total score than those without learning difficulties.

Our reported findings of associations between performance on timed function tests and *DMD* mutation location are congruent with published literature. Chesshyre et al. recently reported associations between *DMD* mutation location, NSAA scores, and intelligence quotient (IQ).^34^ While their stratification based on *DMD* mutation location was different from ours, those with most distal *DMD* mutations showed worse performance compared to those with proximal *DMD* mutations. The mean NSAA total score and mean timed functional tests at age 5 were lowest in research participants with distal *DMD* mutations. Furthermore, NSAA scores were lower by a mean of 2 points in those with intellectual deficit (intelligence quotient two standard deviations below the mean) compared to those with no intellectual deficit. In our study, all participants were corticosteroid‐naive whereas nearly 90% of the cohort reported by Chesshyre et al. received oral corticosteroids.

What are the mechanisms that mediate the association between *DMD* mutation location and the effect of shorter dystrophin isoforms on functional test performance? A lack of task comprehension and attentional influence on functional tests have been reported.^35^, ^36^ While it is clear that “verbal encouragement” improves performance on the 6MWT, this evidence comes from a well‐designed study conducted on older adults.^37^ In our current analysis and earlier publication from the FOR‐DMD trial,^19^ we found only 8% of participants reporting attentional difficulties, though a formal diagnosis of ADHD is not always established prior to age 6. It is possible that mild attentional difficulties are undetected or under‐reported in this age range. Future clinical trials in DMD could consider additional nonmotor attention assessment to understand whether attentional difficulties confound motor assessment.

A neurodevelopmental symptom that has consistently been reported in DMD is SLD. Interestingly, the initial description by Duchenne de Boulogne described expressive language delay in boys with progressive skeletal muscle weakness.^38^ More than 150 years later, the neurobiological underpinnings of SLD in DMD have not been investigated. SLD is an “encompassing” term and refers to both speech disorder (sound and word production) and language disorder (how words are used to communicate). We tried to discern whether the relationships between SLD and functional motor measures in our study were primarily driven by speech versus language disorder by using the age of language acquisition as an index of language disorder. We were not able to discern an association between 6MWD and the age of language acquisition, possibly because only 18 of the 167 participants with data on the timing of language acquisition had a reported speech delay.

While the strengths of our study include the large number of corticosteroid‐naive genetically defined participants with DMD and inclusion of age‐appropriate functional assessments, our study is not without limitations. The first limitation is that we did not perform objective SLD assessments in participants whose parents reported SLD or speech delay. Such objective assessment would provide greater distinction between speech versus language function abnormalities in DMD. Future studies of standardized SLD assessments would address this knowledge gap. The second limitation is that the p values arising from the statistical tests were not adjusted for multiple comparisons as our data analysis is exploratory, and these preliminary findings require confirmation in larger, independent cohorts.

Based on our study findings and precedent in literature, we recommend considering including brief cognitive assessments for future clinical trials in DMD such as the National Institutes of Health Toolbox Cognitive Battery,^39^ digit span,^40^ and standardized neurodevelopmental survey and SLP assessment. Many of these assessments can be conducted within 1 h without too much burden on clinical trial participants. These measured variables can be incorporated as potential covariates in final trial data analyses.

The current landscape of DMD, both from screening and therapeutic standpoint, are advancing rapidly. Although available antisense oligonucleotide therapy and emerging gene therapy are not restorative of CNS pathology in DMD, with anticipated newborn screening being planned for implementation, we forecast that targeting the brain in CNS is going to be the next therapeutic frontier. In sum, our data support more comprehensive neurodevelopmental assessment in DMD in order to *better* serve skeletal health.

## Funding Information

This study was sponsored by The National Institutes of Health (study number U01NS061799) and has also received funding from Telethon Italy, Muscular Dystrophy Association (MDA), and Parent Project Muscular Dystrophy (PPMD). We acknowledge the patient and family organizations including Action Duchenne, Muscular Dystrophy UK, Muscular Dystrophy Canada, and Benni&Co/Parent Project for their promotion of the study. The FOR DMD Steering Committee and the study site Investigators are members of the Muscle Study Group. Michela Guglieri and Kate Bushby are part of the Medical Research Council (UK) and TREAT NMD who also supported the study.

## Conflict of Interest

Dr Guglieri reported receiving grants from Duchenne UK, the European Union's Horizon 2020 program for the Vision‐DMD study (in collaboration with ReveraGen BioPharma Inc), and Sarepta Therapeutics; serving as a consultant to Dyne Therapeutics Inc, Pfizer, and NS Pharma Inc; receiving personal fees from Sarepta Therapeutics; and receiving nonfinancial support from Italfarmaco, Pfizer, ReveraGen BioPharma Inc, and Santhera Pharmaceuticals. Dr McDermott reported receiving grant support from PTC Therapeutics and receiving personal fees from Fulcrum Therapeutics, NeuroDerm Ltd, AstraZeneca, Eli Lilly, Catabasis Pharmaceuticals, Vaccinex Inc, Cynapsus Therapeutics, Neurocrine Biosciences, Voyager Therapeutics, Prilenia Therapeutics, ReveraGen BioPharma Inc, and NS Pharma Inc. Dr Thangarajh reported serving as a consultant to Sarepta Therapeutics and speaker for NS Pharma Inc. Dr Griggs reported serving as a consultant to Strongbridge and Stealth Pharmaceuticals; receiving personal fees from Solid Biosciences and Elsevier; serving as chair of the research advisory committee and is a board member of the American Brain Foundation; and serving on the executive committee of the Muscle Study Group, which receives support for its activities from pharmaceutical companies.

## Author Contributions

M.T., M.M., M.G., R.G.: study concept and design; M.M.: data analysis including statistical evaluation and data interpretation; M.T. prepared the first draft of the manuscript. All authors revised the manuscript.

## Data Availability

The FOR‐DMD study protocol has been previously published.^20^ The FOR‐DMD clinical trial data will be deposited in a publicly available repository and will be released in late 2023.

## References

[1] Duan D , Goemans N , Takeda S , Mercuri E , Aartsma‐Rus A . Duchenne muscular dystrophy. Nat Rev Dis Primers. 2021;7(1):13. doi:10.1038/s41572-021-00248-3 33602943 PMC10557455

[2] Fritschy JM , Schweizer C , Brünig I , Lüscher B . Pre‐ and post‐synaptic mechanisms regulating the clustering of type a gamma‐aminobutyric acid receptors (GABAA receptors). Biochem Soc Trans. 2003;31(Pt 4):889‐892. doi:10.1042/bst0310889 12887328

[3] Fuentes‐Mera L , Rodríguez‐Muñoz R , González‐Ramírez R , et al. Characterization of a novel Dp71 dystrophin‐associated protein complex (DAPC) present in the nucleus of HeLa cells: members of the nuclear DAPC associate with the nuclear matrix. Exp Cell Res. 2006;312(16):3023‐3035. doi:10.1016/j.yexcr.2006.06.002 16824516

[4] Villarreal‐Silva M , Suárez‐Sánchez R , Rodríguez‐Muñoz R , Mornet D , Cisneros B . Dystrophin Dp71 is critical for stability of the DAPs in the nucleus of PC12 cells. Neurochem Res. 2010;35(3):366‐373. doi:10.1007/s11064-009-0064-z 19784870

[5] Constantin B . Dystrophin complex functions as a scaffold for signalling proteins. Biochim Biophys Acta. 2014;1838(2):635‐642. doi:10.1016/j.bbamem.2013.08.023 24021238

[6] Thangarajh M . The Dystrophinopathies. Continuum (Minneap Minn). 2019;25(6):1619‐1639. doi:10.1212/CON.0000000000000791 31794463

[7] Hinton VJ , De Vivo DC , Nereo NE , Goldstein E , Stern Y . Poor verbal working memory across intellectual level in boys with Duchenne dystrophy. Neurology. 2000;54(11):2127‐2132. doi:10.1212/wnl.54.11.2127 10851376 PMC1931422

[8] Wu JY , Kuban KC , Allred E , Shapiro F , Darras BT . Association of Duchenne muscular dystrophy with autism spectrum disorder. J Child Neurol. 2005;20(10):790‐795. doi:10.1177/08830738050200100201 16417872

[9] Ricotti V , Mandy WP , Scoto M , et al. Neurodevelopmental, emotional, and behavioural problems in Duchenne muscular dystrophy in relation to underlying dystrophin gene mutations. Dev Med Child Neurol. 2016;58(1):77‐84. doi:10.1111/dmcn.12922 26365034

[10] Hendriksen JG , Vles JS . Neuropsychiatric disorders in males with duchenne muscular dystrophy: frequency rate of attention‐deficit hyperactivity disorder (ADHD), autism spectrum disorder, and obsessive–compulsive disorder. J Child Neurol. 2008;23(5):477‐481. doi:10.1177/0883073807309775 18354150

[11] Thangarajh M , Spurney CF , Gordish‐Dressman H , et al. Neurodevelopmental needs in young boys with Duchenne muscular dystrophy (DMD): observations from the Cooperative International Neuromuscular Research Group (CINRG) DMD Natural History Study (DNHS). PLoS Curr. 2018;10:ecurrents.md.4cdeb6970e54034db2bc3dfa54b4d987. doi:10.1371/currents.md.4cdeb6970e54034db2bc3dfa54b4d987 30443431 PMC6209412

[12] Caspers Conway K , Mathews KD , Paramsothy P , et al. Neurobehavioral concerns among males with Dystrophinopathy using population‐based surveillance data from the muscular dystrophy surveillance, tracking, and research network. J Dev Behav Pediatr. 2015;36(6):455‐463. doi:10.1097/DBP.0000000000000177 26020585 PMC4497929

[13] Lundy CT , Doherty GM , Hicks EM . Should creatine kinase be checked in all boys presenting with speech delay? Arch Dis Child. 2007;92(7):647‐649. doi:10.1136/adc.2007.117028 17588982 PMC2083798

[14] Cyrulnik SE , Fee RJ , De Vivo DC , Goldstein E , Hinton VJ . Delayed developmental language milestones in children with Duchenne's muscular dystrophy. J Pediatr. 2007;150(5):474‐478. doi:10.1016/j.jpeds.2006.12.045 17452219 PMC1931426

[15] Soim A , Lamb M , Campbell K , et al. A Cross‐sectional study of school experiences of boys with Duchenne and Becker muscular dystrophy. Phys Disabil. 2016;35(2):1‐22. doi:10.14434/pders.v35i2.21765

[16] Hendriksen JGM , Thangarajh M , Kan HE , Muntoni F . ENMC 249th workshop study group. 249th ENMC international workshop: the role of brain dystrophin in muscular dystrophy: implications for clinical care and translational research, Hoofddorp, The Netherlands, November 29th‐December 1st 2019. Neuromuscul Disord. 2020;30(9):782‐794. doi:10.1016/j.nmd.2020.08.357 32912717

[17] Fry AF , Hale S . Processing speed, working memory, and fluid intelligence: evidence for a developmental cascade. Psychol Sci. 1966;7(4):237‐241. doi:10.1111/j.1467-9280.1996.tb00366.x

[18] Desguerre I , Christov C , Mayer M , et al. Clinical heterogeneity of duchenne muscular dystrophy (DMD): definition of sub‐phenotypes and predictive criteria by long‐term follow‐up. PloS One. 2009;4(2):e4347. doi:10.1371/journal.pone.0004347 19194511 PMC2633042

[19] Thangarajh M , Hendriksen J , McDermott MP , et al. Relationships between *DMD* mutations and neurodevelopment in dystrophinopathy. Neurology. 2019;93(17):e1597‐e1604. doi:10.1212/WNL.0000000000008363 31594858 PMC6946466

[20] Guglieri M , Bushby K , McDermott MP , et al. Developing standardized corticosteroid treatment for Duchenne muscular dystrophy. Contemp Clin Trials. 2017;58:34‐39. doi:10.1016/j.cct.2017.04.008 28450193 PMC6279424

[21] Coplan J . Evaluation of the child with delayed speech or language. Pediatr Ann. 1985;14(3):203‐208. doi:10.3928/0090-4481-19850301-05 4000735

[22] Bello L , Kesari A , Gordish‐Dressman H , et al. Genetic modifiers of ambulation in the cooperative international neuromuscular research group Duchenne natural history study. Ann Neurol. 2015;77(4):684‐696. doi:10.1002/ana.24370 25641372 PMC4403971

[23] Bello L , D'Angelo G , Villa M , et al. Genetic modifiers of respiratory function in Duchenne muscular dystrophy. Ann Clin Transl Neurol. 2020;7(5):786‐798. doi:10.1002/acn3.51046 32343055 PMC7261745

[24] Pegoraro E , Hoffman EP , Piva L , et al. SPP1 genotype is a determinant of disease severity in Duchenne muscular dystrophy. Neurology. 2011;76(3):219‐226. doi:10.1212/WNL.0b013e318207afeb 21178099 PMC3034396

[25] Bello L , Morgenroth LP , Gordish‐Dressman H , et al. DMD genotypes and loss of ambulation in the CINRG Duchenne natural history study. Neurology. 2016;87(4):401‐409. doi:10.1212/WNL.0000000000002891 27343068 PMC4977110

[26] Kelley EF , Cross TJ , Synder EM , et al. Influence of β_2_ adrenergic receptor genotype on risk of nocturnal ventilation in patients with Duchenne muscular dystrophy. Respir Res. 2019;20(1):221. doi:10.1186/s12931-019-1200-1 31619245 PMC6796481

[27] Bello L , Flanigan KM , Weiss RB , et al. Association study of exon variants in the NF‐κB and TGFβ pathways identifies CD40 as a modifier of Duchenne muscular dystrophy. Am J Hum Genet. 2016;99(5):1163‐1171. doi:10.1016/j.ajhg.2016.08.023 27745838 PMC5097949

[28] Hogarth MW , Houweling PJ , Thomas KC , et al. Evidence for ACTN3 as a genetic modifier of Duchenne muscular dystrophy. Nat Commun. 2017;8:14143. Published 2017 Jan 31. doi:10.1038/ncomms14143 28139640 PMC5290331

[29] Spitali P , Zaharieva I , Bohringer S , et al. TCTEX1D1 is a genetic modifier of disease progression in Duchenne muscular dystrophy. Eur J Hum Genet. 2020;28(6):815‐825. doi:10.1038/s41431-019-0563-6 31896777 PMC7253478

[30] Weiss RB , Vieland VJ , Dunn DM , Kaminoh Y , Flanigan KM . United Dystrophinopathy project. Long‐range genomic regulators of THBS1 and LTBP4 modify disease severity in duchenne muscular dystrophy. Ann Neurol. 2018;84(2):234‐245. doi:10.1002/ana.25283 30014611 PMC6168392

[31] Flanigan KM , Ceco E , Lamar KM , et al. LTBP4 genotype predicts age of ambulatory loss in Duchenne muscular dystrophy. Ann Neurol. 2013;73(4):481‐488. doi:10.1002/ana.23819 23440719 PMC4106425

[32] McDonald CM , Henricson EK , Abresch RT , et al. Long‐term effects of glucocorticoids on function, quality of life, and survival in patients with Duchenne muscular dystrophy: a prospective cohort study. Lancet. 2018;391(10119):451‐461. doi:10.1016/S0140-6736(17)32160-8 29174484

[33] Markati T , De Waele L , Schara‐Schmidt U , Servais L . Lessons learned from discontinued clinical developments in Duchenne muscular dystrophy. Front Pharmacol. 2021;12:735912. doi:10.3389/fphar.2021.735912 34790118 PMC8591262

[34] Chesshyre M , Ridout D , Hashimoto Y , et al. Investigating the role of dystrophin isoform deficiency in motor function in Duchenne muscular dystrophy. J Cachexia Sarcopenia Muscle. 2022;13(2):1360‐1372. doi:10.1002/jcsm.12914 35083887 PMC8977977

[35] Hoffman EP , McNally EM . Exon‐skipping therapy: a roadblock, detour, or bump in the road? Sci Transl Med. 2014;6(230):230fs14. doi:10.1126/scitranslmed.3008873 PMC4464785 24695683

[36] Shieh PB . Duchenne muscular dystrophy: clinical trials and emerging tribulations. Curr Opin Neurol. 2015;28(5):542‐546. doi:10.1097/WCO.0000000000000243 26280938

[37] Guyatt GH , Pugsley SO , Sullivan MJ , et al. Effect of encouragement on walking test performance. Thorax. 1984;39(11):818‐822. doi:10.1136/thx.39.11.818 6505988 PMC459930

[38] Duchenne GBA . Recherches sur la paralysie musculaire pseudo‐hypertrophique ou paralysie myo‐sclérosique. Arch Gén Méd. 1868;11:5‐25.

[39] Thangarajh M , Kaat AJ , Bibat G , et al. The NIH toolbox for cognitive surveillance in Duchenne muscular dystrophy. Ann Clin Transl Neurol. 2019;6(9):1696‐1706. doi:10.1002/acn3.50867 Erratum in: Ann Clin Transl Neurol. 2019;6(12):2609. 31472009 PMC6764624

[40] Thangarajh M , Elfring GL , Trifillis P , McIntosh J , Peltz SW . Ataluren phase 2b study group. The relationship between deficit in digit span and genotype in nonsense mutation Duchenne muscular dystrophy. Neurology. 2018;91(13):e1215‐e1219. 30135256 10.1212/WNL.0000000000006245 PMC6161548

